# Integration of DNA Methylome and Transcriptome Analysis to Identify Novel Epigenetic Targets in the Acute Kidney Injury–Chronic Kidney Disease Transition

**DOI:** 10.3390/biom15040498

**Published:** 2025-03-29

**Authors:** Xumin Zheng, Xinru Guo, Yuhao Chen, Kaiting Zhuang, Na Gong, Yifei Fu, Yanjun Liang, Yue Xu, Siyang Wang, Wenjuan Wang, Xiangmei Chen, Guangyan Cai

**Affiliations:** 1 Department of Nephrology, First Medical Center of Chinese PLA General Hospital, Nephrology Institute of the Chinese People’s Liberation Army, State Key Laboratory of Kidney Diseases, National Clinical Research Center for Kidney Diseases, Beijing Key Laboratory of Kidney Disease Research, Beijing 100853, China; zhengxumio@126.com (X.Z.);; 2 School of Medicine, Nankai University, Tianjin 300071, China

**Keywords:** epigenetics, DNA methylation, AKI-CKD transition

## Abstract

(1) Background: the epigenetic mechanisms underlying the progression from acute kidney injury (AKI) to chronic kidney disease (CKD) remain poorly understood; (2) Methods: to investigate this process, we conducted genome-wide DNA methylation sequencing to map the epigenetic changes during the AKI-CKD transition in a mouse model. By integrating DNA methylome and transcriptome analyses, we identified genes and signaling pathways regulated by DNA methylation throughout this progression; (3) Results: our analysis identified four candidate genes—*Atp1a3*, *Ncf1*, *Lpl*, and *Slc27a2*—that were regulated by DNA methylation and strongly correlated with kidney disease prognosis. Additionally, we found that the *PPAR* signaling pathways, among others, were implicated in this process. Treatment with DNA methyltransferase inhibitors mitigated fibrosis and improved lipid metabolism in the kidneys during AKI-CKD progression; (4) Conclusions: this study provides the first comprehensive epigenetic map of the AKI-CKD transition. Our findings offer new insights into the epigenetic regulation of kidney disease progression and highlight potential therapeutic targets to prevent the transition from AKI to CKD.

## 1. Introduction

Acute kidney injury (AKI) is a significant global health concern, affecting approximately 13 million individuals annually and contributing to increased mortality and rising healthcare costs [1,2]. Historically, AKI has been viewed as a reversible condition. However, recent large-scale studies have highlighted that survivors of AKI are at a heightened risk of progressing to chronic kidney disease (CKD) [3,4,5]. CKD, which currently affects 9% of the global population, is responsible for 1.2 million deaths each year [6]. Despite its prevalence, there is no pharmacological treatment available to prevent either AKI or its progression to CKD. As such, there is a critical need for research focused on developing reliable biomarkers that can predict the transition from AKI to CKD, guiding monitoring practices, follow-up care, and clinical trial enrollment for potential interventions.

Emerging evidence suggests that epigenetic mechanisms, particularly DNA methylation, play a key role in both AKI and CKD [7,8,9]. Studies have shown that alterations in DNA methylation, especially in the promoter regions of specific genes, are present in AKI, indicating that these changes may serve as potential biomarkers for the condition [10]. Additionally, pharmacological modulation of DNA methylation, using agents like 5-aza-2′-deoxycytidine (5-Aza-dC), as well as demethylation activators such as hydralazine, has been shown to reduce renal fibrosis in CKD models and prevent the progression of AKI to CKD [11,12,13]. These findings suggest that a comprehensive genome-wide DNA methylation map could provide valuable insights into the molecular mechanisms underlying the AKI-CKD transition, identifying key genes and signaling pathways involved.

In this study, we aimed to construct the first DNA methylation map of the AKI-CKD progression by establishing a unilateral renal ischemia/reperfusion injury (UIRI) mouse model of AKI-CKD. Using comprehensive genome-wide DNA methylation and transcriptome sequencing, we identified four genes—*Atp1a3*, *Ncf1*, *Lpl*, and *Slc27a2*—that are regulated by DNA methylation during the AKI-CKD transition. Further analysis, including data from single-cell online databases and the NephroSeq platform, revealed that these genes are significantly associated with kidney disease prognosis. Our findings enhance the understanding of the epigenetic mechanisms driving the progression from AKI to CKD and may offer valuable insights for identifying high-risk patients who are more likely to transition from AKI to CKD.

## 2. Materials and Methods

### 2.1. Animal Models and Experiment Design

All animal care and experimental procedures were approved by the Ethics Committee for Animal Experimentation at the Chinese People’s Liberation Army General Hospital (approval no. 2022-X18-30). Wild-type male C57BL/6 mice (8–9 weeks old, weighing 22–25 g) were obtained from SPF Biotechnology (Beijing, China). To induce unilateral renal ischemia, the left renal pedicle was exposed via a flank incision and clamped for 35 min. 5-Aza-dC (1 mg/kg) was injected into mice intraperitoneally 2 days after UIRI for consecutive 3 days and then every other day. Sham control mice were administered an equivalent volume of saline following the same injection schedule as the treatment group. A total of 66 mice were used in this study, with 6 mice assigned to each experimental group. Detailed procedures are outlined in the Supplementary Materals (Supplementary Methods: Mouse surgery and treatment).

### 2.2. Serum Creatinine and Blood Urea Nitrogen Tests

Blood samples were collected from the inferior vena cava at designated time points. Serum was isolated by centrifuging the samples at 3000 rpm for 15 min at 4 °C. Serum creatinine (Scr) and blood urea nitrogen (BUN) levels were then measured using the picric acid and enzymatic methods, respectively.

### 2.3. Histopathological Examination

Kidney tissues were fixed in 4% formaldehyde for 48 h, then embedded in paraffin and sectioned into 2 μm thick slices. The sections were stained using periodic acid–Schiff (PAS), Masson’s trichrome, or Sirius Red, following standard protocols for each staining method.

### 2.4. Whole-Genome Bisulfite Sequencing (WGBS)

Genomic DNA (gDNA) was extracted from whole kidney tissue using the Gentra Puregene Kit (Qiagen, Germantown, MD, USA) according to the manufacturer’s instructions and stored at −80 °C. For library preparation, 200 ng of gDNA was mixed with 1 ng of unmethylated λ phage DNA and sheared into 200–300 bp fragments using an Ultrasound Generator (Covaris S220, Woburn, MA, USA). After purification, the 3′ ends of the fragmented DNA were repaired by adding adenosine, followed by ligation of TruSeq adapters (Illumina, San Diego, CA, USA). The adapter-ligated DNA was then bisulfite-treated using the ZYMO EZ DNA Methylation-Gold kit (Zymo Research, Irvine, CA, USA) and amplified by PCR. Bisulfite-converted DNA was enriched through multiple PCR cycles using KAPA HiFi HotStart Uracil + DNA polymerase (Kapa Biosystems, Wilmington, MA, USA). Library quality was assessed using a Qubit 2.0 fluorometer (Life Technologies, Carlsbad, CA, USA) and an Agilent 2100 Bioanalyzer (Agilent Technologies Inc., Santa Clara, CA, USA). DNA sequencing was performed on the HiSeq X Ten platform (Illumina, San Diego, CA, USA) according to standard Illumina protocols.

### 2.5. Data Processing and Differential DNA Methylation Analysis

Raw sequencing data were processed using SOAPnuke (v. 2.3) to remove adapter contamination, reads containing more than 1% unknown bases (N), and low-quality reads, defined as those with over 40% of bases having a quality score below 20. Clean reads were aligned to the bisulfite-converted GRCm39 reference genome using Bismark (v. 0.16.1), with default parameters, and duplicates were removed [14]. The methylation information was extracted using Bismark’s tools of bismark_methylation_extractor and coverage2cytosine. The methylation level at each Cytosine–phosphate–Guanine (CpG) site was calculated as the ratio of methylated C reads to the total C reads at that site, multiplied by 100.

### 2.6. Identification of Differentially Methylated Regions

Differentially methylated regions (DMRs) were identified using the DMRcaller R package [15]. For inter-sample DMR analysis, the computeDMRs function from DMRcaller was used. Further details are provided in the Supplementary Methods.

### 2.7. DMR Annotation and Enrichment Analysis

DMRs were annotated using bedtools to associate them with nearby genes or other genomic elements based on their positional relationships [16]. A 1 bp overlap between a DMR and a genomic feature was considered as an association. Gene Ontology (GO) and Kyoto Encyclopedia of Genes and Genomes (KEGG, Kyoto, Japan.) Pathway enrichment analyses were performed on genes associated with DMRs, including those linked to DMR-associated promoters.

### 2.8. RNA Sequencing and Data Processing

Total RNA extraction was performed using the MJzol Animal RNA Isolation Kit (Majorbio, Shanghai, China) following the manufacturer’s protocol, followed by purification with the RNAClean XP Kit (Beckman Coulter, Brea, CA, USA) and RNase-Free DNase Set (QIAGEN, Germantown, MD, USA). The VAHTS Universal V6 RNA-seq Library Prep Kit (Illumina^®^, San Diego, CA, USA) was used for the preparation of transcriptome libraries from the extracted RNA following standard protocols. The concentration and size distribution of the cDNA library were assessed using the Agilent 4200 Bioanalyzer (Agilent Technologies Inc., Santa Clara, CA, USA) prior to sequencing on the Illumina NovaSeq 6000 platform. Raw sequencing data were processed by filtering with Seqtk, and the clean reads were aligned to the reference genome using Hisat2 (version 2.0.4). Gene expression levels were quantified with StringTie (v1.3.3b) and normalized using the trimmed mean of M values (TMM) [17,18,19,20]. Differentially expressed genes (DEGs) were identified based on a False Discovery Rate (FDR) of less than 0.05 and a fold-change greater than 2, using the edgeR software 4.0 [21].

### 2.9. Detection of mRNA

Total RNA from kidney tissue was extracted using TRIzol reagent (Invitrogen, Waltham, MA, USA). mRNA levels were quantified by reverse transcription using HiScript IV RT SuperMix (Vazyme, Nanjing, China), followed by quantitative PCR with the Taq Pro Universal SYBR qPCR Master Mix (Vazyme, Nanjing, China). Gene expression was normalized to GAPDH. Expression values (ΔCt) were calculated using the 2−ΔΔCt method based on triplicate measurements. Primer sequences were provided in Supplementary Table S1.

### 2.10. Bisulfite Pyrosequencing

To validate methylation levels of candidate genes, bisulfite pyrosequencing (BSP) was performed by Shanghai Biotechnology Corporation (Shanghai, China). DNA was extracted from mouse kidney tissue (0.5 μg) using the TIANamp Genomic DNA Kit (TIANGEN, Beijing, China). Bisulfite conversion and DNA purification were conducted using the EZ DNA Methylation-Gold™ Kit (ZYMO, Irvine, CA, USA). BSP primers were designed with PyroMark Assay Design 2.0 software and synthesized by Shanghai Bio-engineering Corporation. PCR amplification was performed using bisulfite-converted DNA as the template, and 10 μL of the PCR product was sequenced on the PyroMark Q48 real-time Quantitative Pyrosequencing Analyzer (Qiagen, Germantown, MD, USA). BSP primer sequences are listed in Supplementary Table S2.

### 2.11. Statistical Analysis

Data were analyzed using Student's t-test for comparisons between two groups, and one-way ANOVA with Tukey’s multiple comparisons test for three or more groups. Pearson’s correlation analysis was used to assess relationships between variables. Statistical analyses were performed using GraphPad Prism 8.0. Data are presented as mean ± SD, and a *p*-value of less than 0.05 was considered statistically significant.

## 3. Results

### 3.1. Establishment of AKI-CKD Animal Model

**Figure 1:**
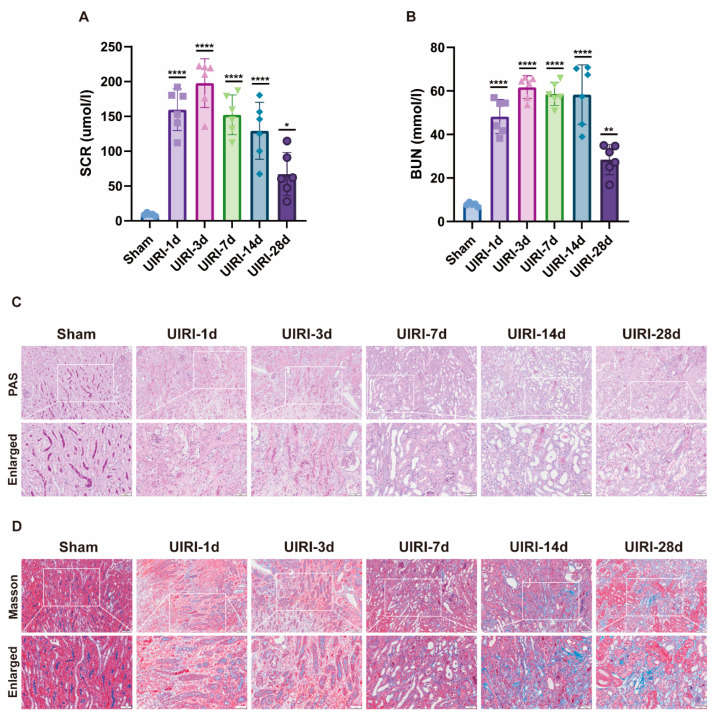
Establishment of the AKI-CKD Animal Model. (**A**,**B**) Biochemical analysis of key renal function markers, including serum creatinine (Scr) and blood urea nitrogen (BUN), during the AKI-CKD transition. (**C**) PAS staining of kidneys from UIRI animals, highlighting histological changes during the AKI-CKD progression. (**D**) Masson’s trichrome staining of UIRI kidneys, illustrating fibrosis development during the AKI-CKD transition. Data are expressed as mean ± SD (*n* = 6); Student’s t-test was used for comparisons between two groups; one-way ANOVA was used for comparisons of three or more groups. * *p* < 0.05, ** *p* < 0.01, **** *p* < 0.0001. scale bar = 100 μm.

An AKI-CKD animal model was established in C57 mice to investigate the transition from acute kidney injury (AKI) to chronic kidney disease (CKD). Biochemical analysis confirmed the successful establishment of the model (Figure 1A,B). Compared to the Sham group, SCR and BUN levels increased rapidly during the early phase of AKI (UIRI-1d, UIRI-3d), gradually declined at subsequent time points, but remained higher than normal levels.

Histological examination using PAS staining (Figure 1C) revealed progressive tubular damage over time. From day 1 to day 7, signs of necrosis, apoptosis, loss of brush border, and tubular dilation were observed. Between days 14 and 28, tubular atrophy, deformation, basement membrane thickening, and interstitial proliferation became evident. Masson’s trichrome staining (Figure 1D) further demonstrated that, from days 1 to 7 post injury, peritubular edema and inflammatory cell infiltration were present. By days 14 to 28, tubular atrophy, interstitial fibrosis, and collagen deposition worsened, reflecting the ongoing progression toward CKD.

### 3.2. The Overall Landscape of DNA Methylation in the AKI-CKD Transition

To investigate the DNA methylation landscape during the progression from AKI to CKD, we analyzed kidney tissues from six time points across the AKI-CKD continuum using Whole Genome Bisulfite Sequencing (WGBS). A total of 18 samples were processed. Raw sequencing data were cleaned by removing contaminants, sequencing adapters, and low-quality reads. After filtering, each sample yielded an average of 85.09 Gbp of high-quality bases. Detailed WGBS data for each sample are provided in Supplementary Table S3.

**Figure 2:**
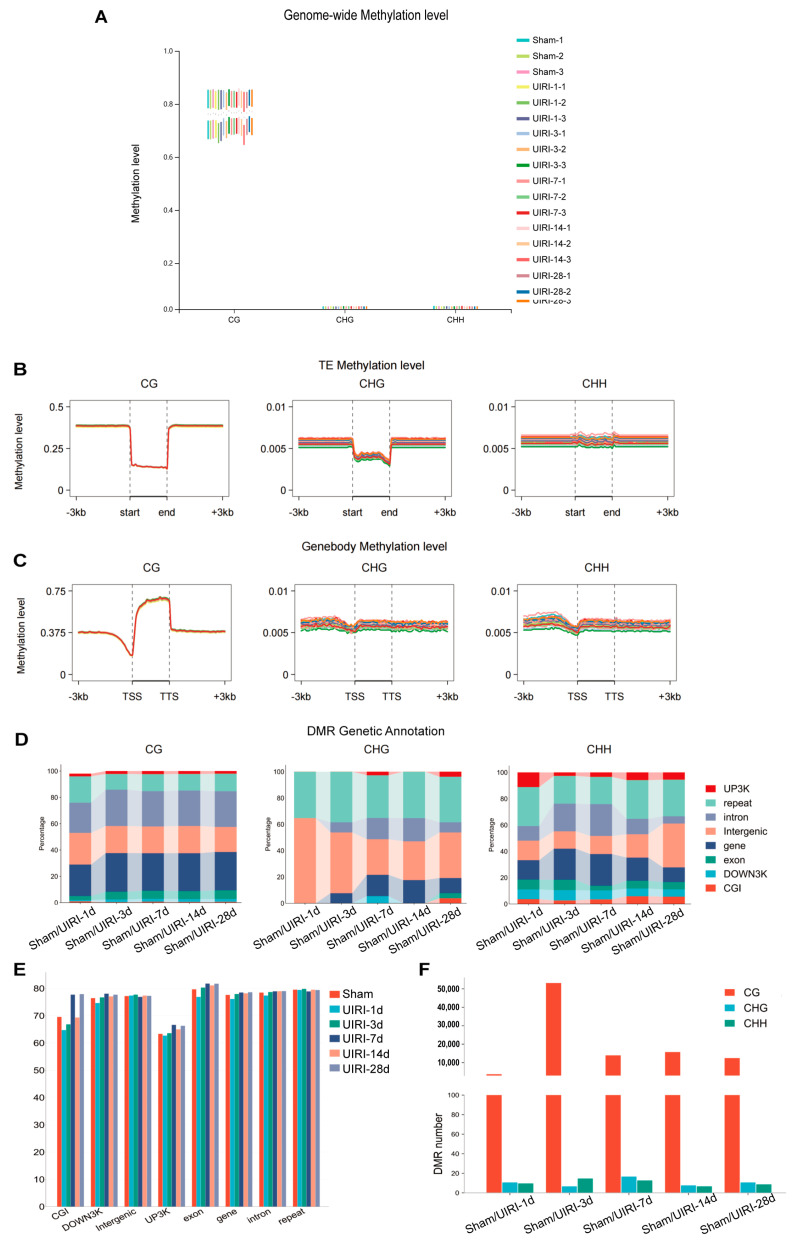
Global DNA Methylome Landscape in the AKI-CKD Transition. (**A**) Box plot illustrating the genome-wide methylation levels of CG, CHG, and CHH contexts during the AKI-CKD transition. (**B**) Line plot showing the methylation levels of transposable elements (TEs) in CG, CHG, and CHH contexts throughout the AKI-CKD progression. (**C**) Line plot displaying gene body methylation levels in CG, CHG, and CHH contexts during the AKI-CKD transition. (**D**) Link bar plot depicting the genetic annotation of differentially methylated regions (DMRs) in CG, CHG, and CHH contexts. (**E**) Bar plot representing the methylation levels of DMRs in the CG context during the AKI-CKD transition. (**F**) Bar plot showing the number of DMRs in the CG, CHG, and CHH contexts during the AKI-CKD transition.

We assessed DNA methylation at three types of sites (CG, CHG, CHH) across the genome for each sample. CpG islands exhibited a significantly higher proportion of methylation compared to CHG and CHH regions (Figure 2A). In comparison to the Sham group (71.87%), the CG methylation level at UIRI-1d was slightly reduced (71.59%), but increased levels were observed in subsequent time points: UIRI-3d (72.33%), UIRI-7d (73.05%), UIRI-14d (72.54%), and UIRI-28d (73.11%).

Methylation levels in transposon regions were also evaluated (Figure 2B). At UIRI-1d, CG methylation decreased slightly compared to the Sham group (14.10% to 13.90%), but no significant changes were observed in later groups: UIRI-3d (14.09%), UIRI-7d (14.00%), UIRI-14d (14.11%), and UIRI-28d (14.07%).

Furthermore, we examined methylation in gene regions (Figure 2C). At UIRI-1d, CG methylation showed a slight reduction (62.12%) compared to the Sham group (63.09%). However, methylation levels increased in subsequent groups: UIRI-3d (63.45%), UIRI-7d (63.27%), UIRI-14d (63.58%), and UIRI-28d (63.48%).

Lastly, we quantified the number of differentially methylated regions (DMRs) across the AKI-CKD progression (Figure 2F). The UIRI-3d group exhibited the highest number of DMRs, with 53,352 regions identified. The methylation patterns within genomic functional elements in these DMRs are shown in Figure 2E, and their distribution changes are presented in Figure 2D.

### 3.3. DMR Landscape During AKI-CKD Progression

**Figure 3:**
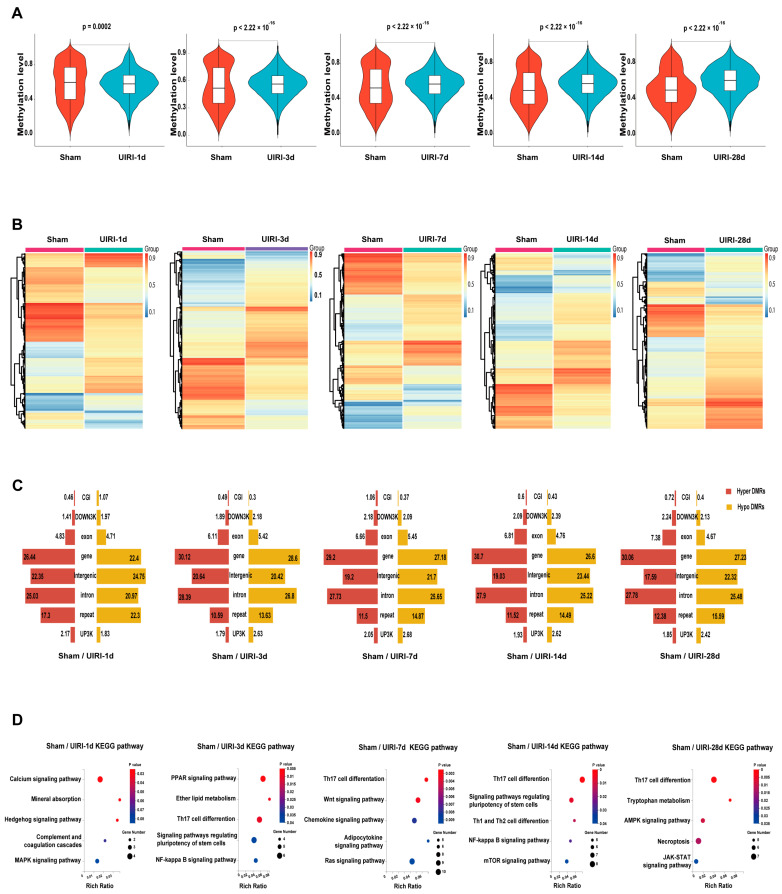
DMR Landscapes in the AKI-CKD Transition. (**A**) Violin plots showing the average methylation levels of differentially methylated regions (DMRs) during the AKI-CKD transition. (**B**) Heatmaps illustrating the methylation patterns of DMRs in the AKI-CKD transition. (**C**) Butterfly plot displaying the ratio of hypermethylated and hypomethylated DMRs across various genomic regions, including CG-island, downstream 3kb (down3k), exon, gene body, intergenic, intron, repeat elements, and promoter (UP3K). (**D**) The top five KEGG pathways associated with genes in the promoter and CG-island regions of DMRs.

We analyzed the average methylation levels of differentially methylated regions (DMRs) across all groups (Figure 3A). Compared to the Sham group, the UIRI-1d group showed a significant decrease in DMR methylation (*p* < 0.05), while methylation levels in the later groups (UIRI-3d, UIRI-7d, UIRI-14d, and UIRI-28d) were significantly higher (*p* < 0.05). These findings indicate that DNA methylation in DMRs initially decreases during the early phase (1d) of AKI but increases as the disease progresses towards CKD.

The heatmaps showed an overall changes in DNA methylation in the progression of AKI to CKD (Figure 3C). Analysis of DMRs revealed that most DMRs were in the coding regions or intergenic areas of genes (Figure 3C).

To further investigate the biological implications of these changes, we performed KEGG pathway analysis on genes located in the promoter and CpG island regions of the DMRs in each group. The top five most relevant pathways for each group are shown in Figure 3D.

### 3.4. Integrated Analysis of DNA Methylome and Transcriptome to Identify Methylation-Driven Genes in AKI-CKD Transition

**Figure 4:**
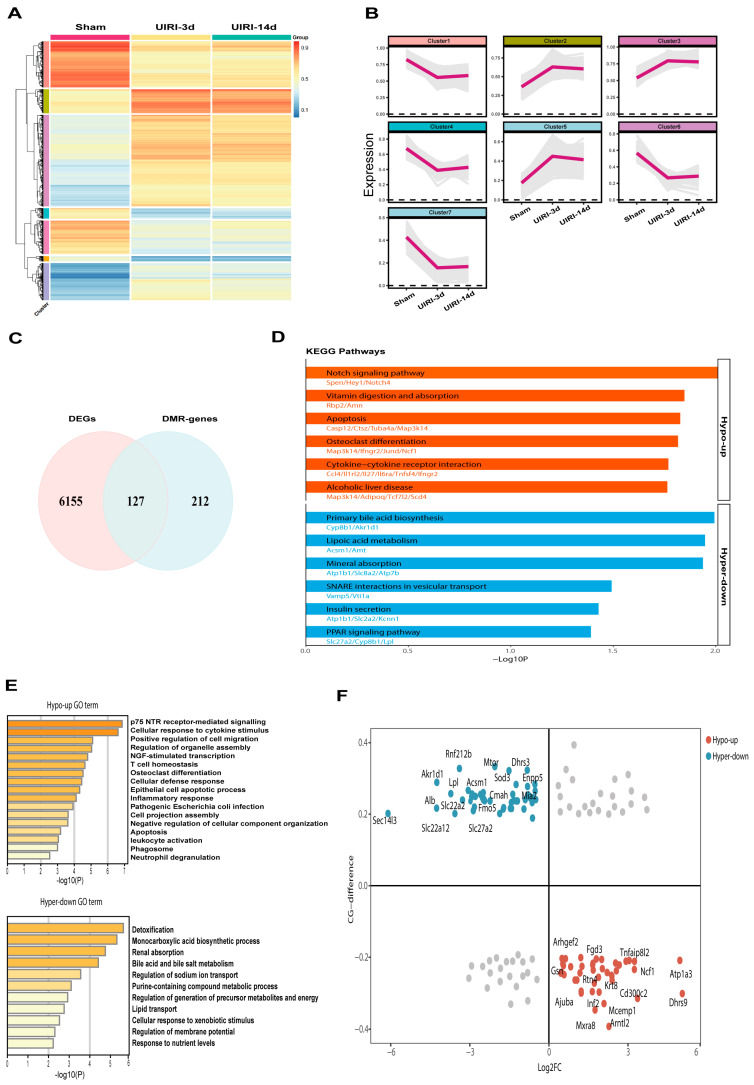
Integrated Analysis of WGBS and Transcriptome Identifies Methylation-Driven Genes in the AKI-CKD Transition. (**A**) Heatmap showing the methylation levels of common differentially methylated regions (DMRs) during the AKI-CKD transition. (**B**) Line plot depicting the clustering of DMRs based on their methylation levels and variation patterns throughout the AKI-CKD progression. (**C**) Venn diagram illustrating the overlap between differentially expressed genes (DEGs) and DMR-associated genes in the AKI-CKD transition. (**D**) KEGG pathway analysis of common genes exhibiting either hypermethylation or hypomethylation. (**E**) Gene Ontology (GO) enrichment analysis of common genes with hypermethylation or hypomethylation. (**F**) Starburst plot comparing differential DNA methylation and gene expression. Red circles represent genes that are significantly hypomethylated and upregulated in the AKI-CKD transition, while blue circles indicate genes that are significantly hypermethylated and downregulated.

To identify candidate genes regulated by methylation during the progression from AKI to CKD, we selected representative groups: Sham, UIRI-3d, and UIRI-14d. We first obtained the differentially methylated regions (DMRs) between Sham and UIRI-3d, and Sham and UIRI-14d, and then intersected these two DMRs, which resulted in a total of 7847 common DMRs. These DMRs were then subjected to clustering analysis (Figure 4A). Based on their methylation levels and trends, they were classified into seven distinct clusters (Figure 4B).

Next, we performed RNA sequencing (RNA-seq) on kidney tissues from the Sham, UIRI-3d, and UIRI-14d groups. A total of 7870 differentially expressed genes (DEGs) were identified between the Sham and UIRI-3d groups, while 8875 DEGs were found between the Sham and UIRI-14d groups. The intersection of the DEGs from both comparisons revealed 6282 common DEGs. We then focused on genes whose promoter regions or CpG islands overlapped with the identified DMRs, yielding 127 common genes potentially regulated by DNA methylation during AKI-CKD progression (Figure 4C). KEGG and GO pathway analyses were performed on these 127 genes, and the top-ranked pathways and GO terms are presented in Figure 4D,F.

Since DNA hypermethylation generally silences gene expression, while hypomethylation promotes gene expression, we performed quadrant analysis based on the expression levels and methylation status of these 127 genes. Genes located at the blue points, which displayed low expression and hypermethylation, were classified as silenced by DNA methylation. In contrast, genes at the red points, with high expression and hypomethylation, were considered activated by DNA demethylation (Figure 4E). Based on the inverse correlation between promoter methylation and gene expression, the genes in the blue and red circles were identified as methylation-driven genes potentially involved in the AKI-CKD transition. A total of 84 genes met these criteria, shown in Supplementary Table S4.

### 3.5. Identification of Atp1a3, Ncf1, Lpl, and Slc27a2 as Key Candidates in AKI-CKD Progression

**Figure 5:**
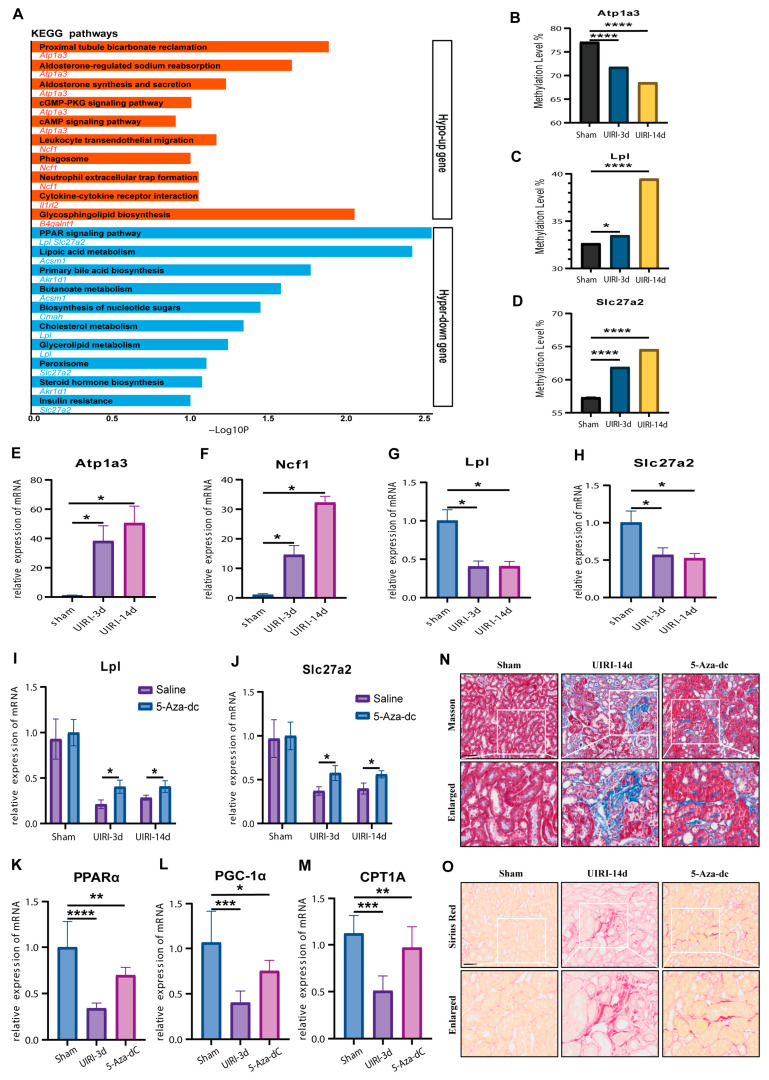
Identification of Methylation-Driven Genes in the AKI-CKD Transition: *Atp1a3*, *Ncf1*, *Lpl*, and *Slc27a2* as Major Candidates. (**A**) KEGG pathway analysis of methylation-driven genes with an absolute Log2 fold change (Log2FC) value ≥ 2 during the AKI-CKD transition. (**B**–**D**) Methylation levels of *Atp1a3*, *Lpl*, and *Slc27a2* in the AKI-CKD transition, validated by targeted bisulfite sequencing. (**E**–**H**) mRNA expression levels of *Atp1a3*, *Ncf1*, *Lpl*, and *Slc27a2* in kidney tissues from the AKI-CKD transition, measured by qRT-PCR. (**I**,**J**) mRNA expression levels of *Lpl* and *Slc27a2* in kidney tissues treated with 5-Aza-dC, assessed by qRT-PCR. (**K**–**M**) mRNA expression levels of *PPARα*, *PGC-1α*, and *CPT1A* in kidney tissues treated with 5-Aza-dC, measured by qRT-PCR. (**N**) Masson’s trichrome staining showing collagen deposition in kidney tissues treated with 5-Aza-dC. (**O**) Sirius Red staining depicting collagen accumulation in kidney tissues treated with 5-Aza-dC. Data are expressed as mean ± SD (*n* = 6); Student’s t-test was used for comparisons between two groups; one-way ANOVA was used for comparisons of three or more groups. * *p* < 0.05, ** *p* < 0.01, *** *p* < 0.001, **** *p* < 0.0001. scale bar = 50 μm.

We conducted KEGG pathway enrichment analysis on genes with an absolute Log2FC value ≥ 2 (Figure 5A). Among the genes exhibiting hypomethylation, *Atp1a3* and *Ncf1* were enriched in more pathways, primarily related to immune and metabolic processes. Conversely, among genes exhibiting hypermethylation, *Lpl* and *Slc27a2* were enriched in more pathways, such as *PPAR* signaling pathway and lipoic acid metabolism. These findings highlight the distinct roles of these genes in the transition from AKI to CKD.

To validate the methylation changes in these four candidate genes, we performed targeted bisulfite sequencing. Unfortunately, the PCR probes for *Ncf1* failed to capture the DMRs. However, *Atp1a3*, *Lpl*, and *Slc27a2* displayed methylation changes that were consistent with the whole-genome bisulfite sequencing data, further confirming the methylation alterations observed during the AKI-CKD transition (Figure 5B–D).

Next, we used qPCR to assess the expression of these candidate genes at the mRNA level. The expression patterns (Figure 5E–H) were consistent with those observed in the transcriptomic analysis, confirming the correlation between DNA methylation changes and gene expression. To further investigate the role of DNA methylation, we treated UIRI model mice with the demethylating agent 5-Aza-dC. The treatment led to a significant increase in the expression of *Lpl* and *Slc27a2* compared to the UIRI group (*p* < 0.05) (Figure 5I,J), supporting the hypothesis that these genes are regulated by DNA methylation.

Given that KEGG analysis pointed to lipid metabolism pathways, we next examined several lipid metabolism-related molecules by qPCR. The results (Figure 5K–M) showed that *PPARα*, *PGC-1α*, and *CTP1A* were significantly reduced in the UIRI group compared to the Sham group (*p* < 0.05). However, the 5-Aza-dC treatment restored the expression of these genes, indicating a recovery of lipid metabolism processes (*p* < 0.05).

Finally, considering that impaired lipid metabolism contributes to fibrosis in CKD progression, we assessed renal tissue fibrosis. The 5-Aza-dC treatment significantly reduced fibrosis compared to the UIRI group (Figure 5N,O). These findings suggest that inhibiting DNA hypermethylation during AKI-CKD progression can restore lipid metabolism and mitigate fibrosis, providing insights into potential therapeutic strategies.

### 3.6. Correlation Between Candidate Genes and Kidney Disease Prognosis

**Figure 6:**
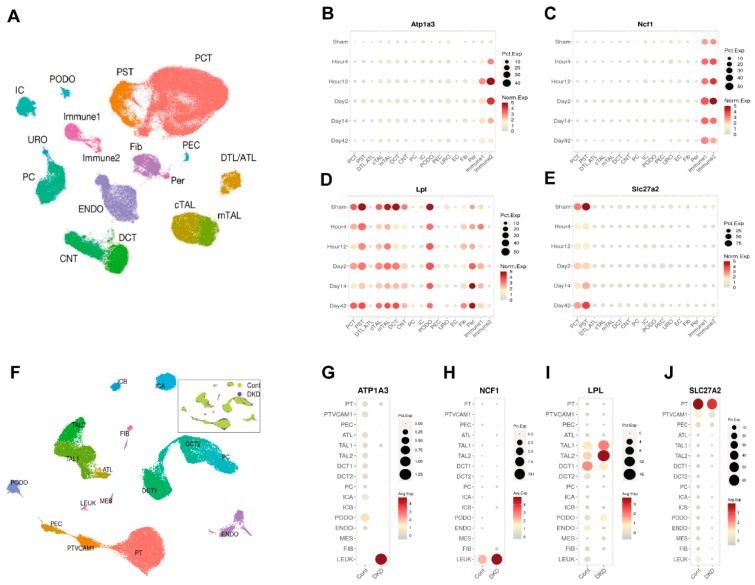
Single-Cell RNA Sequencing Reveals the Localization and Expression of *Atp1a3*, *Ncf1*, *Lpl*, and *Slc27a2* in Kidney. (**A**) UMAP plot depicting distinct cell types in mouse kidneys following ischemia–reperfusion injury (IRI). (**B**–**E**) Dot plots illustrating the localization and expression patterns of *Atp1a3*, *Ncf1*, *Lpl*, and *Slc27a2* in the mouse IRI kidney. (**F**) UMAP plot showing distinct cell types in human kidneys with diabetic kidney disease (DKD). (**F**–**J**) Dot plots demonstrating the localization and expression patterns of *Atp1a3*, *Ncf1*, *Lpl*, and *Slc27a2* in human DKD kidney tissue.

We analyzed the renal single-cell RNA sequencing data from the KIT database (https://www.humphreyslab.com/SingleCell/, accessed on 20 October 2024.) to investigate the localization and expression patterns of the candidate genes in both mouse models and human kidney diseases. In the mouse ischemia–reperfusion injury (IRI) model (Figure 6A–E), *Atp1a3*, and *Ncf1* were primarily expressed in immune cells, with their levels increasing after injury. *Lpl* was predominantly found in tubular epithelial cells, where its expression decreased following injury. *Slc27a2* was localized to proximal renal tubular cells, and its expression also reduced after injury. In the human Diabetic Kidney Disease (DKD) tissues(Figure 6F–J), *Atp1a3*, and *Ncf1* were mainly expressed in leukocytes, with their expression levels elevated in the DKD group. *Lpl* was localized to distal tubular epithelial cells, and its expression decreased in the DKD group. *Slc27a2* was found in proximal renal tubular cells, showing a similar decrease in expression in the DKD group. These patterns in both mouse models and human kidney tissues align with the trends observed in the initial sequencing data.

**Figure 7:**
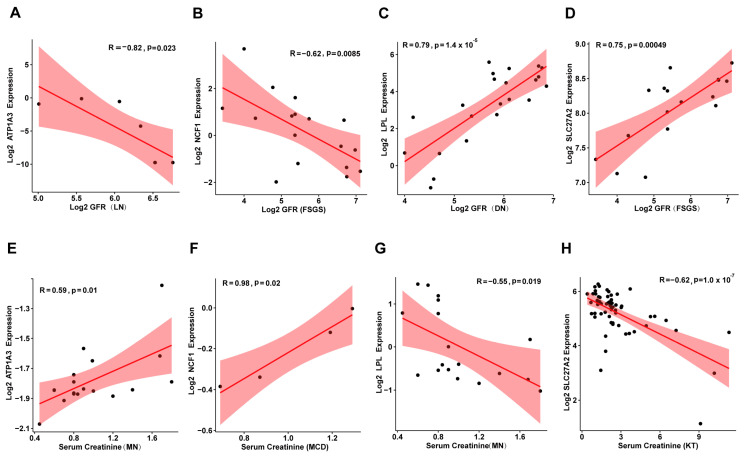
Correlation of *Atp1a3*, *Ncf1*, *Lpl*, and *Slc27a2* Expression with GFR and Serum Creatinine in Human Kidney Diseases. (**A**–**D**) Scatter plots showing the correlation between *Atp1a3*, *Ncf1*, *Lpl*, and *Slc27a2* expression and glomerular filtration rate (GFR) in human kidney diseases. (**E**–**H**) Scatter plots illustrating the relationship between *Atp1a3*, *Ncf1*, *Lpl*, and *Slc27a2* expression and serum creatinine levels in human kidney diseases. MN: Membranous Nephropathy; FSGS: Focal Segmental Glomerulosclerosis; DN: Diabetic Nephropathy; MCD: Minimal Change Disease; KT: Kidney Transplantation.

To further explore the potential role of these genes in kidney disease prognosis, we analyzed clinical data, including glomerular filtration rate (GFR), serum creatinine levels, and gene expression from the Nephroseq database (https://www.nephroseq.org, accessed on 28 October 2024.). The results revealed that *Atp1a3* and *Ncf1* were negatively correlated with GFR (Figure 7A,B) and positively correlated with serum creatinine levels (Figure 7E,F). In contrast, *Lpl* and *Slc27a2* exhibited the opposite trends, showing positive correlations with GFR (Figure 7C,D) and negative correlations with serum creatinine (Figure 7G,H). These findings suggest that *Atp1a3* and *Ncf1* may play a role as pathogenic factors in kidney disease progression, while *Lpl* and *Slc27a2* could act as protective factors. However, further experimental studies are needed to fully understand the underlying mechanisms through which these genes influence kidney disease.

## 4. Discussion

Previous studies have focused on DNA methylation in isolated acute kidney injury or chronic kidney disease [8,10,22], but few have addressed DNA methylation changes during the AKI-CKD transition, with limited renal-wide investigations. In this study, we applied whole-genome bisulfite sequencing to map the DNA methylation landscape in the kidney throughout the AKI-CKD progression. At the genomic level, we observed only slight, non-significant changes in overall DNA methylation, which aligns with findings by Huang et al. [23]. However, at the level of differentially methylated regions (DMRs), we noted a decrease in methylation during the early stages of AKI-CKD progression (UIRI-1d), followed by a gradual increase in later stages. This suggests a dynamic shift in DNA methylation throughout the AKI-CKD transition. Our findings contrast with Zhao et al. [24], who reported a significant decrease in CpG methylation within 24 h and 7 days after ischemia–reperfusion injury IRI. These differences may be due to variations in sequencing methods and AKI-CKD models.

By integrating WGBS data with transcriptomics, we identified 84 candidate genes potentially regulated by DNA methylation during AKI-CKD progression. From these, we highlighted four key genes for further investigation. The DNA methylation patterns and expression levels of these genes were validated in kidney tissue from AKI-CKD models using bisulfite sequencing PCR and quantitative PCR. In addition, single-cell sequencing data revealed the renal localization of these genes during the AKI-CKD progression. We found that *Atp1a3* and *Ncf1*, which exhibited low DNA methylation and high expression, were predominantly expressed in immune cells, whereas *Lpl* and *Slc27a2*, with high DNA methylation and low expression, were primarily localized to renal tubular epithelial cells.

*Atp1a3* encodes the Na^+^, K^+^-ATPase α3 subunit, an essential component of the sodium–potassium pump, which helps maintain cellular functions. While limited research has explored *Atp1a3* in kidney disease, its expression in immune cells suggests a role in immune activation and regulation [25]. *Ncf1*, encoding a subunit of the NADPH oxidase complex, is involved in neutrophil function, inflammation, and oxidative stress, all of which are critical in kidney disease pathogenesis. Studies show that *Ncf1* knockout can alleviate kidney injury and fibrosis [26,27,28]. The *Lpl* gene (lipoprotein lipase) encodes lipoprotein lipase (LPL), an enzyme that catalyzes the hydrolysis of triglycerides (TG) into free fatty acids and glycerol, which can be absorbed by tissues for energy metabolism. Mutations in *Lpl* lead to lipid deposition in the kidney and a decline in renal function [29,30]. The *SLC27A2* (*Solute Carrier Family 27 Member 2*) encodes a protein known as Long-Chain Fatty Acid Transporter 2 (LFACT2), which is responsible for transporting long-chain fatty acids into cells. Its dysfunction can impair fatty acid metabolism, exacerbating kidney injury and fibrosis [31,32,33].

To validate the role of DNA hypermethylation in AKI-CKD, we treated AKI-CKD model mice with the DNA methylation inhibitor 5-Aza-dC. This treatment led to restored expression of the hypermethylated genes *Lpl* and *Slc27a2*. Moreover, we observed increased expression of key enzymes involved in fatty acid metabolism, including *PPARα*, *PGC-1α*, and *CPT1A*, alongside reduced kidney fibrosis during AKI-CKD progression. These findings are consistent with previous studies, which reported that azacitidine treatment in acute myeloid leukemia enhances fatty acid oxidation and improves fatty acid metabolism [34,35]. Additionally, DNA methyltransferase inhibitors like decitabine have been shown to improve metabolic profiles in animal models, further supporting our findings [36]. However, further studies are needed to confirm whether 5-Aza-dC can mitigate lipid metabolic disorders and alleviate kidney damage and fibrosis by reversing hypermethylation in renal tissues.

Preclinical studies suggest that targeting epigenetic mechanisms holds promise for treating AKI and related kidney diseases. Several epigenetic drugs have already been used in other diseases, such as histone deacetylase (HDAC) inhibitors like suberoylanilide hydroxamic acid (SAHA), vorinostat, for skin T-cell lymphoma, Valproic Acid (VPA) for epilepsy and bipolar disorder, and DNA methylation inhibitors like decitabine for myelodysplastic syndromes and acute myeloid leukemia [37]. However, no such drugs have been tested in clinical trials for kidney diseases. A major challenge with epigenetic drugs is their potential nonspecificity, which can lead to global, non-gene-specific changes across the genome and various organs. To address this, emerging CRISPR-Cas9-based approaches offer a promising alternative. This technique allows for precise and targeted DNA demethylation at specific genomic sites without the need for epigenetic-modifying enzymes. By evaluating the functional consequences of demethylation in specific contexts, this technology could open new avenues for epigenetic therapies [38,39,40].

## 5. Conclusions

In conclusion, our study highlights the important role of epigenetic changes in the progression from AKI to CKD. We identified DNA methylation alterations in the *Atp1a3*, *Ncf1*, *Lpl*, and *Slc27a2* genes, which occur in distinct kidney cell types during the AKI-CKD transition. These changes may influence kidney injury and repair processes, likely through the regulation of fatty acid metabolism. Our findings offer valuable insights into the epigenetic and transcriptomic shifts associated with AKI-CKD, providing a foundation for early identification of patients at high risk for progression from AKI to CKD. Additionally, these results suggest potential targets for therapeutic intervention.

## Data Availability

The original contributions presented in this study are included in the article/Supplementary Materials. Further inquiries can be directed to the corresponding author.

## Supplementary Materials

The following supporting information can be downloaded at: https://www.mdpi.com/article/10.3390/biom15040498/s1, Figure S1: Venn diagram showing the intersection of DMRs between the Sham vs. UIRI-3d, and Sham vs. UIRI-14d; Figure S2: Venn diagram showing the intersection of DEGs between the Sham vs. UIRI-3d, and Sham vs. UIRI-14d; Figure S3: Volcano plot showing the distribution of 7870 DEGs between the Sham group and the UIRI-3d group. Calculation parameters: |log2FC|≥1,Qvalue≤0.05; Figure S4: Volcano plot showing the distribution of 7,870 DEGs between the Sham group and the UIRI-3d group. Calculation parameters: |log2FC|≥1,Qvalue≤0.05; Figure S5: Starburst plot showing differential DNA methylation versus differential gene expression between Sham and UIRI-3d; Table S1: Real-time qPCR primer sequences; Table S2: BSP primer sequences; Table S3: Statistical results of methylated cytosines in different contexts (WGBS); Table S4: Candidate genes that may be regulated by DNA methylation during the AKI-CKD progression; Table S5: Quality statistics of filtered reads (RNA sequencing); Table S6: Alignment of clean reads to reference genome using HISAT( RNA sequencing); Table S7: Alignment of clean reads to reference genome using Bowtie2 (RNA sequencing).

## Abbreviations

The following abbreviations are used in this manuscript:

5-Aza-dC	5-Aza-2′-deoxycytidine
AKI	Acute Kidney Injury
ATL	Ascending Thin Limb
ATL	Ascending Thin Limb
Atp1a3	ATPase Na^+^/K^+^ transporting subunit alpha 3
BSP	Bisulfite Pyrosequencing
BUN	Blood Urea Nitrogen
cATL	Cortical Ascending Thin Limb
CG	Cytosine-Guanine dinucleotide
CHG	Cytosine-Homologous base-Guanine dinucleotide
CHH	Cytosine-Homologous base-Homologous base dinucleotide
CKD	Chronic Kidney Disease
CNT	Connecting Tubule
CpG	Cytosine-phosphate-Guanine
CTP1A	Carnitine palmitoyltransferase 1A
DCT	Distal Convoluted Tubule
DEGs	Differentially Expressed Genes
DKD	Diabetic Kidney Disease
DMRs	Differentially Methylated Regions
DTL	Descending Thin Limb
DTL	Descending Thin Limb
EC	Endothelial Cells
ENDO	Endothelial Cells
Fib	Fibroblasts
GFR	Glomerular Filtration Rate
GO	Gene Ontology
HDAC	Histone Deacetylase
IC	Intercalated Cells
ICA	Intercalated Cell Type A
ICB	Intercalated Cell Type B
Immune	Immune Cells
KEGG	Kyoto Encyclopedia of Genes and Genomes
LEUK	Leukocytes
Lpl	Lipoprotein lipase
mATL	Medullary Ascending Thin Limb
MES	Mesangial Cells
Ncf1	Neutrophil cytosolic factor 1
PAS	Periodic Acid-Schiff
PC	Principal Cells
PCT	Proximal Convoluted Tubule
PCT	Proximal Convoluted Tubule
PEC	Parietal Epithelial Cells
Per	Pericytes
PGC-1α	Peroxisome proliferator-activated receptor gamma coactivator 1-alpha
PODO	Podocytes
PPAR	Peroxisome Proliferator-Activated Receptor
PST	Proximal Straight Tubule
PST	Proximal Straight Tubule
PT	Proximal Tubule
PTVCAM1	Proximal Tubule VCAM1+ Cells
SAHA	Suberoylanilide Hydroxamic Acid
Scr	Serum creatinine
Slc27a2	Solute carrier family 27 member 2
TAL1	Thick Ascending Limb 1
TAL2	Thick Ascending Limb 2
UIRI	Unilateral Renal Ischemia/Reperfusion Injury
URO	Urothelial Cells
VPA	Valproic Acid
WGBS	Whole Genome Bisulfite Sequencing
