# Supplementary material for: Integration of DNA Methylome and Transcriptome Analysis to Identify Novel Epigenetic Targets in the Acute Kidney Injury–Chronic Kidney Disease Transition
Source: Biomolecules. 2025 Mar 29;15(4):498. doi: 10.3390/biom15040498 (PMC12024732; doi:10.3390/biom15040498)
Supplement: Supplementary file 1 [file biomolecules-15-00498-s001.zip › biomolecules-3489141-supplementary.pdf]

Integration of DNA methylome and transcriptome analysis to identify novel epigenetic targets in the AKI-CKD transition

1. Supplementary Figures

Supplementary Figure S1

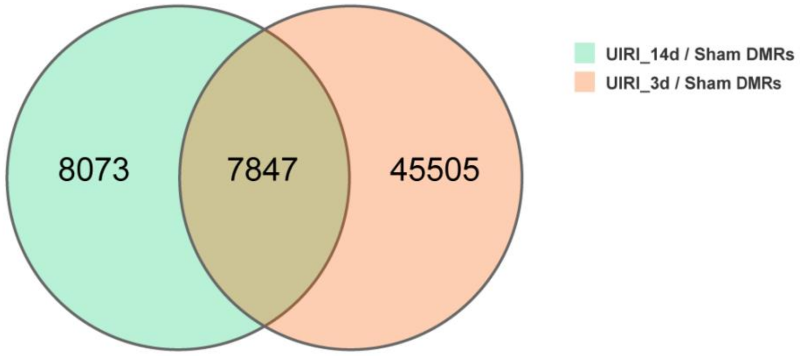

Supplementary Figure S1. Venn diagram showing the intersection of DMRs between the Sham vs. UIRI-3d, and Sham vs. UIRI-14d.

Supplementary Figure S2

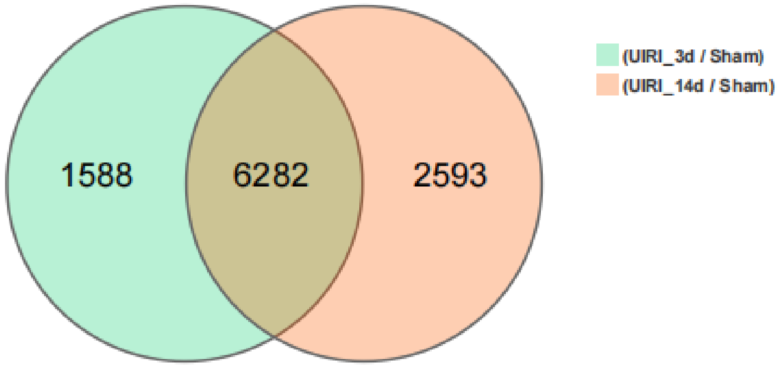

Supplementary Figure S2. Venn diagram showing the intersection of DMGs between the Sham vs. UIRI-3d, and Sham vs. UIRI-14d.

Supplementary Figure S3

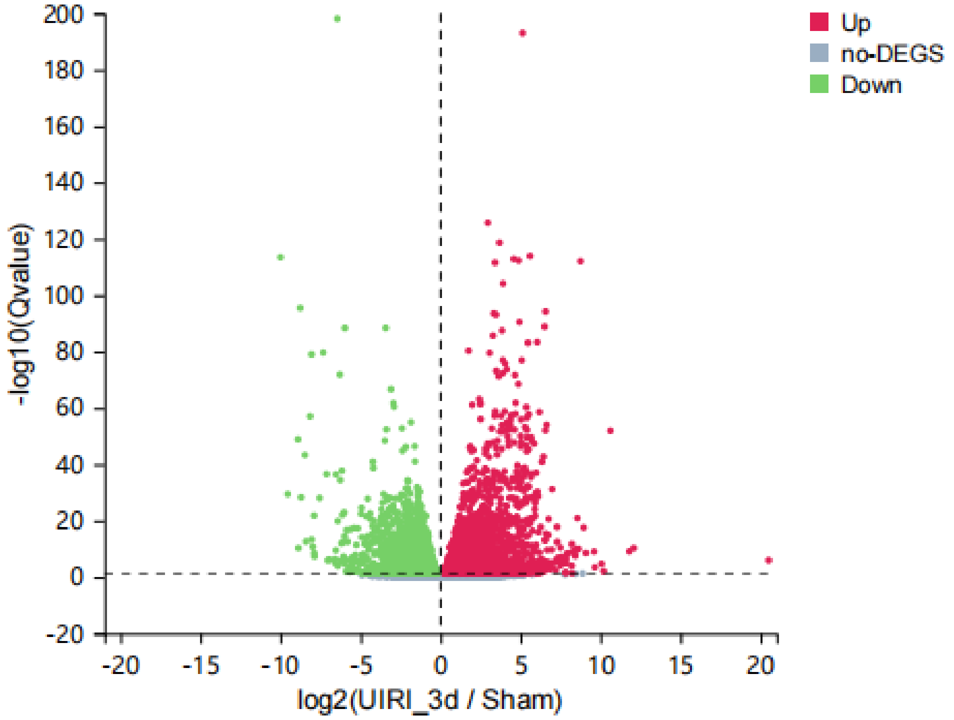

Supplementary Figure S3. Volcano plot showing the distribution of 7,870 DEGs between the Sham group and the UIRI-3d group. Calculation parameters: |log2FC|≥1,Qvalue≤0.05.

Supplementary Figure S4

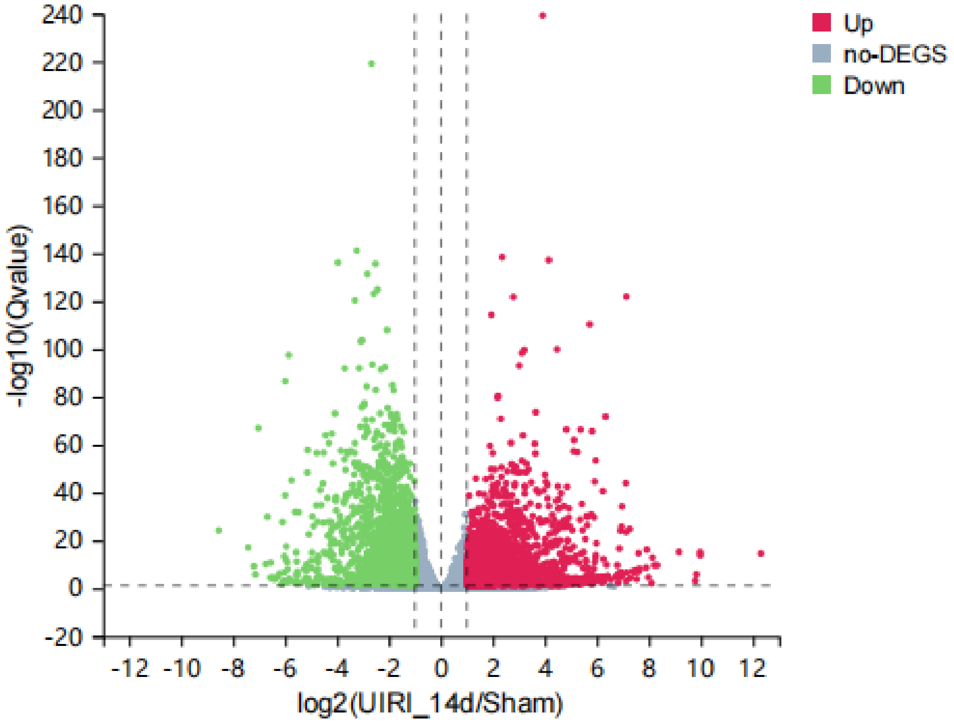

Supplementary Figure S4. Volcano plot showing the distribution of 7,870 DEGs between the Sham group and the UIRI-3d group. Calculation parameters: |log2FC|⩾ 1,Qvalue⩽0.05.

Supplementary Figure S5

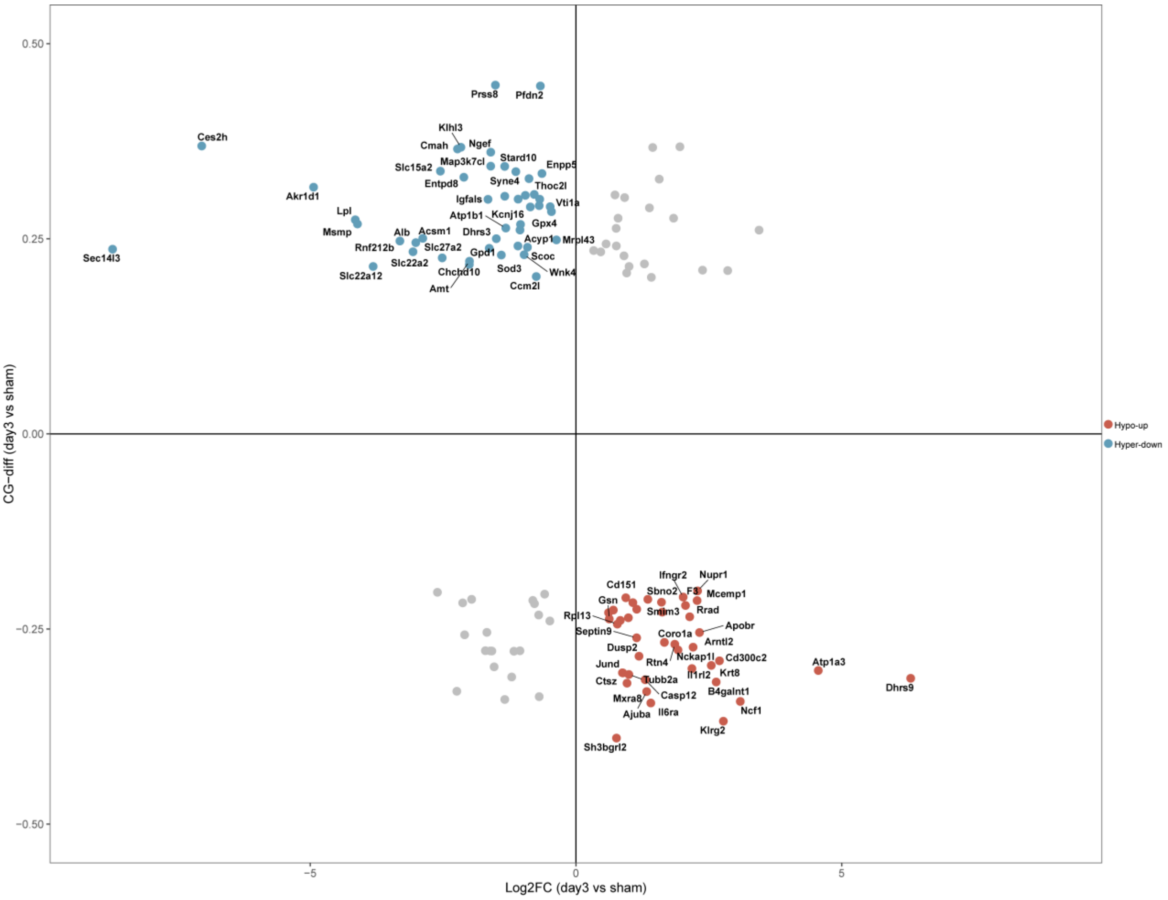

Supplementary Figure S5. Starburst plot showing differential DNA methylation versus differential gene expression between Sham and UIRI-3d.

2. Supplementary Methods

Mouse surgery and treatment

In brief, following anesthetization of the mice, the dorsal hair above the kidney was shaved and the left renal pedicle vessel was then exposed and clamped using a nontraumatic vascular clamp (Harvard Apparatus, Holliston, MA) for 35 min; during this process, the body temperature of the mice was strictly maintained at 37°C. In

sham control mice, the kidneys were exposed but the renal pedicle vessels were not clamped.

Mice were euthanized at 1 day (UIRI-1 d), 3 day (UIRI-3 d), 7 day (UIRI-7 d), 14 day (UIRI-14 d) and 28 day (UIRI-28 d). To evaluate the function of the UIRI kidney, the right kidney was removed on the day before euthanizing. If there are obvious lesions such as renal cysts or subcapsular bleeding, these samples should be excluded.

DMR identification

(1) The calculation parameters of DMR between samples:

regions = REGIONS

context = CONTEXT

method = "bins"

binSize=200

test = "fisher"

ValueThreshold = 0.05

minCytosinesCount = 5

minReadsPerCytosine = 5

minProportionDifference = 0.2

minGap = 0

cores = 1

(2) The calculation parameters of DMR inter-samples:

condition = CONDITIONS

regions = REGIONS

context = CONTEXT

method = "bins"

binSize=200

test = "betareg"

pValueThreshold = 0.05

minCytosinesCount = 5

minReadsPerCytosine = 5

minProportionDifference = 0.2

minGap = 0

cores = 1

Enrichment analysis

GO functional enrichment analysis identifies the Gene Ontology (GO) terms that are significantly enriched in the candidate genes compared to the entire gene background of the species, thus revealing which biological functions are significantly associated with the candidate genes. This analysis first maps all candidate genes to the various entries in the Gene Ontology database (http://www.geneontology.org/), calculates the number of genes for each entry, and then applies a hypergeometric test to identify GO terms that are significantly enriched in the candidate genes compared to the background genes of the species.

The p-values are computed using the basic R function 'phyper' (https://stat.ethz.ch/R-manual/R-devel/library/stats/html/Hypergeometric.html). Subsequently, the p-values undergo multiple testing correction using the 'qvalue' package (https://bioconductor.org/packages/release/bioc/html/qvalue.html). Finally, GO terms with a corrected q-value (P-value) ≤ 0.05 are considered significantly enriched in the candidate genes. KEGG Pathway enrichment analysis further aids in understanding the biological functions of genes. KEGG Pathway is one of the most widely used public databases for pathway-related information. Pathway significance enrichment analysis uses KEGG Pathways as units and applies a hypergeometric test to identify pathways that are significantly enriched in the candidate genes compared to the entire genomic background. This analysis follows the same methodology as the GO functional enrichment analysis; for detailed procedures, please refer to the description of GO enrichment analysis above. Finally, pathways with a Q-value ≤ 0.05 are defined as significantly enriched in the differentially expressed genes. Through pathway significance enrichment, the key biochemical metabolic and signaling pathways involved in the candidate genes can be identified.

Clustering and Visualization of DMRs

(1) Clustering and Heatmap Visualization

We used the pheatmap package in R to perform hierarchical clustering and visualize the DMRs. Code for Heatmap:

```
library(pheatmap)
# Heatmap visualization
p <- pheatmap(DMR,
        border_color = "white",    # Set border color
        cluster_rows = TRUE,    # Perform row clustering
        cutree_rows = 7,    # Cut row dendrogram into 7 clusters
        cluster_cols = FALSE,    # No column clustering
        gaps_col = c(1, 2),    # Add gaps between columns
        show_rownames = FALSE,    # Hide row names
        show_colnames = FALSE,    # Hide column names
        angle_col = 45,    # Rotate column labels by 45 degrees
        fontsize_row = 12,    # Set row label font size
        fontsize_col = 12,    # Set column label font size
        legend_breaks = c(0.1, 0.5, 0.9),    # Set legend breaks
        annotation_col = annotation_col,    # Add column annotations
        annotation_colors = ann_colors,    # Define annotation colors
        annotation_row = annotation_row,    # Add row annotations
)
```

(2) Extracting Cluster Information

After clustering, we extracted the cluster assignments and analyzed the DMRs within each cluster:

Code for Extracting Clusters:

```
# Extract cluster assignments
row_cluster <- cutree(p$tree_row, k = 7)    # Assign rows to 7 clusters
# Reorder data based on clustering
```

```
newOrder <- DMR4[p$tree_row$order, ]
newOrder$Cluster <- row_cluster[match(rownames(newOrder), names(row_cluster))]
colnames(newOrder)[ncol(newOrder)] <- "Cluster"
# Add "Cluster" prefix to cluster numbers
newOrder$Cluster <- paste0("Cluster", newOrder$Cluster)
# Save clustered data
# write.table(newOrder, "expr_DE.pheatmap.cluster.txt", sep = "\t", quote = FALSE,
row.names = TRUE, col.names = TRUE)
```

(3) Visualization of Cluster Trends

To visualize the trends of DMRs within each cluster, we used ggplot2 to plot the average methylation patterns:

Code for Trend Plot:

```
library(ggplot2)
library(reshape2)
# Convert data to long format
data_new <- melt(newOrder, id.vars = c("gene", "Cluster"))
# Plot cluster trends
ggplot(data_new, aes(variable, value, group = gene)) +
  geom_line(color = "gray90", size = 0.8) +   # Individual DMR trends
  geom_hline(yintercept = 0, linetype = 2) +   # Add reference line
  stat_summary(aes(group = 1), fun = mean, geom = "line", linewidth = 1.2, color =
"#c51b7d") +   # Mean trend line
  facet_wrap(~Cluster, nrow = 3, scales = "free_y", labeller = label_both) +   # Split
by cluster
  labs(x = "Stage", y = "Methylation Level") +   # Axis labels
  theme_bw() +   # Use black-and-white theme
  theme(
    panel.grid.major = element_blank(),
    panel.grid.minor = element_blank(),
```

```
    strip.background = element_rect(color = "black", size = 1, linetype = "solid", fill = "lightblue"),
    panel.border = element_rect(color = "black", size = 1),
    axis.text = element_text(size = 8, face = "bold"),
    axis.text.x = element_text(angle = 60, hjust = 1),
    strip.text = element_text(size = 10, face = "bold"),
    panel.spacing = unit(0.5, "lines")
  )
```

3. Supplementary Tables

Table S1. Real-time qPCR primer sequences

**Gene**		**primer sequences(5'- 3')**
Mouse Atp1a3	Forward	GGTGTGGGTATCATCTCTGAGG
Mouse Atp1a3	Reverse	CGTCAATCTGCTCCGAGGTGAA
Mouse Ncf1	Forward	GCTGACTACGAGAAGAGTTCGG
Mouse Ncf1	Reverse	CCTCGCTTTGTCTTCATCTGGC
Mouse Lpl	Forward	GCGTAGCAGGAAGTCTGACCAA
Mouse Lpl	Reverse	AGCGTCATCAGGAGAAAGGCGA
Mouse Slc27a2	Forward	TTCAACAGCGGAGACCTCCTGA
Mouse Slc27a2	Reverse	CCACGATGTCAGCGACTTCTGT
Mouse CPT1A	Forward	GGCATAAACGCAGAGCATTCCTG
Mouse CPT1A	Reverse	CAGTGTCCATCCTCTGAGTAGC
Mouse PPARa	Forward	ACCACTACGGAGTTCACGCATG
Mouse PPARa	Reverse	GAATCTTGCAGCTCCGATCACAC
Mouse PGC-1α	Forward	GAATCAAGCCACTACAGACACCG
Mouse PGC-1α	Reverse	CATCCCTCTTGAGCCTTTCGTG

Table S2. BSP primer sequences

Gene		primer sequences(5’- 3’)
Mouse Atp1a3	Forward	TGTGGTGGTAGGTTTGTTATT
Mouse Atp1a3	RBIO	ACACTCCCTATACCTCTCAAAT
Mouse Atp1a3	Reverse	ATATAAGGAGAATTTATTTTTAGAT
Mouse Lpl	Forward	ATAGGAGTTTGATTTGGAGTTTATG
Mouse Lpl	RBIO	TAACCTTAATCAAATACCCCAATTCTC
Mouse Lpl	Reverse	TTGATTTGGAGTTTATGTGA
Mouse Slc27a2	Forward	TGTGTAGAGGATGGTATTGTATAAG
Mouse Slc27a2	RBIO	CAACTCTTTAAAATACCCATTAATACCAA
Mouse Slc27a2	Reverse	GTATAAGAAAAGTGGGTTTTGA

Table S3. Statistical results of methylated cytosines in different contexts (WGBS).

Sample Name	Total Clean Reads	Total Mapping Reads	Total Mapping Ratio (%)	Unique Mapping Reads	Unique Mapping Ratio (%)	Methylation Conversion (%)	mCG Methylation Level (%)	mCHG Methylation Level (%)	mCHH Methylation Level (%)
**Sham_1**	567104990	444880256	78.45	417451868	73.61	99.51	71.51	0.63	0.66
**Sham_2**	567188882	443693788	78.23	416406898	73.42	99.51	72.07	0.59	0.61
**Sham_3**	567338082	452129428	79.69	424130652	74.76	99.52	72.04	0.58	0.59
**UIRI_1d_1**	567342150	437946062	77.19	407788668	71.88	99.52	71.07	0.59	0.60
**UIRI_1d_2**	567271254	440755856	77.7	410845096	72.42	99.51	71.47	0.59	0.60
**UIRI_1d_3**	567299890	436797612	77	406161576	71.6	99.51	72.23	0.61	0.63
**UIRI_3d_1**	567425970	443790228	78.21	416252120	73.36	99.51	72.40	0.56	0.56
**UIRI_3d_2**	567256996	446306448	78.68	420247392	74.08	99.52	71.49	0.57	0.58
**UIRI_3d_3**	567079012	432446626	76.26	405012976	71.42	99.53	73.09	0.54	0.55
**UIRI_7d_1**	567186450	438152184	77.25	411428418	72.54	99.51	72.60	0.56	0.57
**UIRI_7d_2**	567229522	416766028	73.47	390847414	68.9	99.51	73.65	0.56	0.57
**UIRI_7d_3**	564625642	421639058	74.68	395781296	70.1	99.49	72.91	0.57	0.58
**UIRI_14d_1**	567162752	452109720	79.71	424549672	74.85	99.52	71.39	0.56	0.57
**UIRI_14d_2**	567813452	428084716	75.39	402044038	70.81	99.47	73.62	0.60	0.61

**UIRI_14d_3**	567284602	452713284	79.8	423991996	74.74	99.51	72.62	0.59	0.60
**UIRI_28d_1**	567480308	455333402	80.24	427646330	75.36	99.48	72.51	0.62	0.63
**UIRI_28d_2**	567523250	429744152	75.72	403991194	71.18	99.46	73.18	0.63	0.64
**UIRI_28d_3**	567362916	419391868	73.92	394264188	69.49	99.46	73.64	0.64	0.65

Table S4. Candidate genes that may be regulated by DNA methylation during the AKI-CKD progression.

Methylation-Gene expression	Gene symbol	Log2FC	Qvalue Sham / UIRI (gene expression)	CG difference	Qvalue Sham / UIRI (CG difference)
Hyper-down	Sec14l3	-8.7187266	4.86E-29	0.00552248	0.236639
Hyper-down	Akr1d1	-4.9365023	7.04E-15	1.18E-05	0.316092
Hyper-down	Lpl	-4.152021	1.17E-20	1.47E-07	0.274283
Hyper-down	Msmp	-4.1110428	0.0024176	0.000973	0.268932
Hyper-down	Slc22a12	-3.8158781	0.00144453	0.04119368	0.214487
Hyper-down	Rnf212b	-3.3126516	0.00965168	0.00895026	0.247104
Hyper-down	Slc22a2	-3.0661531	4.33E-13	0.00140429	0.233322
Hyper-down	Alb	-3.0133914	4.72E-06	0.01002337	0.245031
Hyper-down	Acsm1	-2.8825089	2.35E-10	0.00536074	0.250554
Hyper-down	Slc15a2	-2.5511357	1.64E-28	0.00140697	0.336612
Hyper-down	Slc27a2	-2.5166848	3.88E-05	0.00438348	0.225511
Hyper-down	Cmah	-2.2286477	2.91E-14	3.59E-06	0.365041
Hyper-down	Klhl3	-2.1648226	3.18E-15	2.25E-05	0.367232
Hyper-down	Entpd8	-2.1095169	7.85E-05	1.21E-10	0.328826

Hyper-down	Chchd10	-2.0059339	4.27E-09	0.000935	0.217047
Hyper-down	Amt	-2.0031978	1.54E-27	0.04394257	0.221211
Hyper-down	Igfals	-1.6552291	3.15E-05	2.32E-10	0.300529
Hyper-down	Gpd1	-1.6315827	8.55E-06	0.01490743	0.237758
Hyper-down	Map3k7cl	-1.603818	4.29E-07	4.74E-07	0.343004
Hyper-down	Ngef	-1.6031808	4.40E-05	1.85E-05	0.360911
Hyper-down	Prss8	-1.5156214	4.05E-05	0.00026	0.446846
Hyper-down	Dhrs3	-1.4985836	7.63E-21	0.02361023	0.250103
Hyper-down	Sod3	-1.4038152	0.01641871	0.03417873	0.229159
Hyper-down	Stard10	-1.3406907	6.19E-09	0.000103	0.342724
Hyper-down	Slc25a33	-1.3393308	1.12E-07	0.00309149	0.304486
Hyper-down	Atp1b1	-1.3197829	1.31E-05	0.00237634	0.263705
Hyper-down	Syne4	-1.1309471	5.18E-05	0.0254284	0.335968
Hyper-down	Acyp1	-1.0914732	3.14E-08	0.00328388	0.24075
Hyper-down	Ctbs	-1.0878232	1.56E-09	8.55E-05	0.300752
Hyper-down	Gpx4	-1.0526456	6.06E-08	0.000123	0.261196
Hyper-down	Kcnj16	-1.0429171	1.27E-05	0.000162	0.268375
Hyper-down	Wnk4	-0.9746613	1.00E-05	3.50E-05	0.229557
Hyper-down	Hsd17b7	-0.9523742	2.80E-05	5.49E-06	0.305582
Hyper-down	Scoc	-0.912104	2.74E-11	0.000506	0.239128
Hyper-down	Thoc2l	-0.8836386	0.000532	9.54E-05	0.327052
Hyper-down	Tmem135	-0.8584572	5.81E-10	9.48E-11	0.29078
Hyper-down	Zfp503	-0.7842001	2.76E-08	1.46E-05	0.306744
Hyper-down	Ccm2l	-0.746793	0.00301909	6.57E-08	0.201612
Hyper-down	Gatad1	-0.6917164	4.28E-06	1.37E-06	0.292446
Hyper-down	Mia2	-0.6813038	1.98E-05	0.01306937	0.300644

Hyper-down	Pfdn2	-0.6692134	4.53E-05	2.00E-14	0.445753
Hyper-down	Enpp5	-0.6397606	1.40E-06	0.00242991	0.333572
Hyper-down	Unc119b	-0.4849964	0.00117186	3.18E-06	0.2911
Hyper-down	Vti1a	-0.4607631	6.61E-05	0.00312982	0.284758
Hyper-down	Mrpl43	-0.3673142	0.02648704	8.78E-05	0.248538
Hypo-up	Dhrs9	5.06041783	0.00360264	4.78E-10	-0.30084
Hypo-up	Atp1a3	4.96717982	6.21E-17	0.000898	-0.208592
Hypo-up	Cd300c2	3.36290844	2.26E-14	1.28E-11	-0.314305
Hypo-up	Ncf1	3.24221231	1.74E-15	0.000935	-0.233718
Hypo-up	Casp12	3.04678994	7.08E-32	7.06E-05	-0.208909
Hypo-up	Dusp2	2.923851	1.31E-12	0.01078422	-0.210932
Hypo-up	Nckap1l	2.66919694	1.63E-12	5.72E-06	-0.221335
Hypo-up	Rrad	2.55890406	3.66E-15	0.00306167	-0.237458
Hypo-up	Il1rl2	2.52824332	3.06E-19	0.000143	-0.215957
Hypo-up	Apobr	2.39743988	1.71E-07	4.07E-06	-0.263704
Hypo-up	B4galnt1	2.39010567	2.09E-39	0.000169	-0.252061
Hypo-up	Klrg2	2.37653424	6.43E-13	1.06E-22	-0.250114
Hypo-up	Arntl2	2.27818376	1.20E-11	8.72E-34	-0.392197
Hypo-up	Coro1a	2.20953636	1.87E-09	0.000112	-0.242255
Hypo-up	Mcemp1	2.09086953	0.00119521	2.62E-06	-0.328675
Hypo-up	Il6ra	2.02713405	6.75E-07	0.00644237	-0.231635
Hypo-up	Krt8	1.90563783	2.09E-13	0.000804	-0.29681
Hypo-up	Sbno2	1.88200696	2.45E-60	3.47E-07	-0.25565
Hypo-up	F3	1.79745446	1.01E-28	5.87E-06	-0.204583
Hypo-up	Mxra8	1.75051663	3.52E-14	3.02E-06	-0.346385
Hypo-up	Ptprs	1.72333126	5.83E-28	3.34E-09	-0.271864

Hypo-up	Nupr1	1.68057378	0.000193	0.00201093	-0.235493
Hypo-up	Smim3	1.67053265	6.38E-14	1.26E-27	-0.216583
Hypo-up	Ifngr2	1.65984332	5.38E-40	0.00184064	-0.22115
Hypo-up	Fgd3	1.25538819	4.19E-20	0.000188	-0.225118
Hypo-up	Ajuba	1.22588661	2.69E-08	8.71E-09	-0.299368
Hypo-up	Rtn4	1.22337114	2.15E-21	4.39E-05	-0.294289
Hypo-up	Arhgef40	1.20973477	6.78E-08	1.13E-11	-0.200904
Hypo-up	Tubb2a	1.17896746	1.36E-08	7.18E-17	-0.243421
Hypo-up	Map3k14	1.06251385	3.57E-09	4.77E-12	-0.263301
Hypo-up	Septin9	0.82650657	2.09E-13	0.02878686	-0.226321
Hypo-up	Rps15a-ps5	0.80122648	2.15E-05	9.81E-12	-0.320887
Hypo-up	Arhgef2	0.62965126	3.39E-13	0.03625511	-0.203907
Hypo-up	Ctsz	0.57573227	0.00104436	7.66E-11	-0.248295
Hypo-up	Cd151	0.52620033	0.000359	0.03351888	-0.218295
Hypo-up	Gsn	0.49684699	2.79E-06	0.00583927	-0.203381
Hypo-up	Asap1	0.49629541	0.000522	3.35E-06	-0.243717
Hypo-up	Jund	0.45451804	0.03875835	0.000101	-0.244059
Hypo-up	Rpl13	0.44122161	0.000117	0.000458	-0.263187

Table S5. Quality statistics of filtered reads（RNA sequencing）.

Sample	Total Raw Reads (M)	Total Clean Reads (M)	Total Clean Bases (Gb)	Clean Reads Q20 (%)	Clean Reads Q30 (%)	Clean Reads Ratio (%)
**Sham_1**	48.93	45.3	6.79	97.44	92.38	92.56

**Sham_2**	48.93	44.51	6.68	97.44	92.47	90.97
**Sham_3**	48.93	44.59	6.69	97.34	92.17	91.13
**UIRI_1_1**	48.93	44.16	6.62	97.35	92.26	90.24
**UIRI_1_2**	48.93	44.91	6.74	97.39	92.26	91.79
**UIRI_1_3**	48.93	44.45	6.67	97.56	92.7	90.83
**UIRI_3_1**	48.93	44.81	6.72	97.43	92.41	91.57
**UIRI_3_2**	48.93	45.13	6.77	97.46	92.5	92.23
**UIRI_3_3**	48.93	45.39	6.81	97.65	93	92.75
**UIRI_7_1**	48.93	44.85	6.73	97.55	92.74	91.65
**UIRI_7_2**	48.93	44.89	6.73	97.41	92.36	91.73
**UIRI_7_3**	48.93	45.08	6.76	97.43	92.42	92.13
**UIRI_14_1**	48.93	44.84	6.73	97.47	92.56	91.64
**UIRI_14_2**	47.19	44.57	6.69	96.57	90.76	94.45
**UIRI_14_3**	47.19	44.32	6.65	96.61	90.83	93.92
**UIRI_28_1**	47.19	44.75	6.71	96.18	89.72	94.84
**UIRI_28_2**	47.19	44.88	6.73	96.66	91.08	95.11
UIRI_28_3	47.19	45.02	6.75	96.62	90.91	95.42

Table S6. Alignment of clean reads to reference genome using HISAT（RNA sequencing）.

Sample	Total Clean Reads (M)	Total Mapping(%)	Uniquely Mapping(%)
**Sham_1**	45.3	98.63	88.32
**Sham_2**	44.51	98.26	88.49
**Sham_3**	44.59	98.11	88.18
**UIRI_1_1**	44.16	98.45	87.84
**UIRI_1_2**	44.91	98.64	90.24
**UIRI_1_3**	44.45	98.61	87.92
**UIRI_3_1**	44.81	98.51	91.91
**UIRI_3_2**	45.13	98.52	90.88
**UIRI_3_3**	45.39	98.65	90.94
**UIRI_7_1**	44.85	98.41	91.32
**UIRI_7_2**	44.89	98.2	91.04
**UIRI_7_3**	45.08	98.51	91.19
**UIRI_14_1**	44.84	98.31	91.51
**UIRI_14_2**	44.57	98.38	91.71

**UIRI_14_3**	44.32	98.31	91.99
**UIRI_28_1**	44.75	98.54	91.11
**UIRI_28_2**	44.88	98.57	89.84
**UIRI_28_3**	45.02	98.65	89.03

Table S7. Alignment of clean reads to reference genome using Bowtie2（RNA sequencing）.

Sample	Total Clean Reads (M)	Total Mapping(%)	Uniquely Mapping(%)
**Sham_1**	45.3	69.75	65.84
**Sham_2**	44.51	71.17	67.33
**Sham_3**	44.59	69.8	66.13
**UIRI_1_1**	44.16	70.52	64.89
**UIRI_1_2**	44.91	77.12	71.01
**UIRI_1_3**	44.45	70.06	64.61
**UIRI_3_1**	44.81	79.27	73.77
**UIRI_3_2**	45.13	76.33	71.35
**UIRI_3_3**	45.39	77.8	72.03
**UIRI_7_1**	44.85	76.71	71.55

**UIRI_7_2**	44.89	75.56	70.31
**UIRI_7_3**	45.08	76.48	71.18
**UIRI_14_1**	44.84	76.36	70.88
**UIRI_14_2**	44.57	75.49	70.51
**UIRI_14_3**	44.32	76.08	71.27
**UIRI_28_1**	44.75	72.93	68.19
**UIRI_28_2**	44.88	71.01	66.47
**UIRI_28_3**	45.02	69.43	65.34
